# Peripheral and spinal 5-HT receptors participate in cholestatic itch and antinociception induced by bile duct ligation in rats

**DOI:** 10.1038/srep36286

**Published:** 2016-11-08

**Authors:** Bin Tian, Xue-Long Wang, Ya Huang, Li-Hua Chen, Ruo-Xiao Cheng, Feng-Ming Zhou, Ran Guo, Jun-Cheng Li, Tong Liu

**Affiliations:** 1 Department of General Surgery, The Second Affiliated Hospital of Soochow University, Suzhou, 215004, China; 2 Jiangsu Key Laboratory of Translational Research and Therapy for Neuro-Psycho-Diseases, Institute of Neuroscience, Soochow University, Suzhou, Jiangsu, 215123, China; 3 Jiangsu Key Laboratory of Preventive and Translational Medicine for Geriatric Diseases, School of Public Health, Soochow University, Suzhou, 215123, China; 4 Department of Anesthesiology, The First Affiliated Hospital of Soochow University, Suzhou, 215006, China

## Abstract

Although 5-HT has been implicated in cholestatic itch and antinociception, two common phenomena in patients with cholestatic disease, the roles of 5-HT receptor subtypes are unclear. Herein, we investigated the roles of 5-HT receptors in itch and antinociception associated with cholestasis, which was induced by common bile duct ligation (BDL) in rats. 5-HT-induced enhanced scratching and antinociception to mechanical and heat stimuli were demonstrated in BDL rats. 5-HT level in the skin and spinal cord was significantly increased in BDL rats. Quantitative RT-PCR analysis showed 5-HT_1B_, 5-HT_1D_, 5-HT_2A_, 5-HT_3A_, 5-HT_5B_, 5-HT_6_, and 5-HT_7_ were up-regulated in peripheral nervous system and 5-HT_1A_, 5-HT_1F_, 5-HT_2B_, and 5-HT_3A_ were down-regulated in the spinal cord of BDL rats. Intradermal 5-HT_2_, 5-HT_3_, and 5-HT_7_ receptor agonists induced scratching in BDL rats, whereas 5-HT_3_ agonist did not induce scratching in sham rats. 5-HT_1A_, 5-HT_2_, 5-HT_3_, and 5-HT_7_ agonists or antagonists suppressed itch in BDL rats. 5-HT_1A_ agonist attenuated, but 5-HT_1A_ antagonist enhanced antinociception in BDL rats. 5-HT_2_ and 5-HT_3_ agonists or antagonists attenuated antinociception in BDL rats. Our data suggested peripheral and central 5-HT system dynamically participated in itch and antinociception under cholestasis condition and targeting 5-HT receptors may be an effective treatment for cholestatic itch.

Itch (pruritus) is an unpleasant somatic sensation that elicits a desire to scratch^1^. Acute itch serves as a self-protective mechanism against potential harmful environmental irritants or parasites^2^. However, chronic itch is a debilitating symptom that arises from many systemic disorders, such as dermatologic diseases (e.g. atopic dermatitis and psoriasis), chronic kidney failure, chronic liver diseases (e.g. cholestasis), infections, and hematologic diseases^3^,^4^. Scratching transiently relieves acute itch^5^, but has limited effects on chronic itch and paradoxically evokes itch-scratch-itch cycle. Chronic itch disrupts sleep and substantially reduces the quality of life of patients^1^. Antihistamines are often clinically prescribed for treating allergy itch; however, they are inefficient for many aforementioned chronic itch conditions^4^. Although the recent discovery for itch-specific neural pathway^6^,^7^,^8^, novel itch mediators and receptors^9^,^10^,^11^,^12^, greatly improves our understanding on acute itch^2^, the pathogenesis of chronic itch associated with systemic disorders remains enigmatic.

Cholestasis is defined as diminished delivery of bile into the intestine resulting either from a functional defect at hepatocyte level or obstruction at the bile duct level of any cause, such as primary biliary cirrhosis (PBC), primary sclerosing cholangitis (PSC), intrahepatic cholestasis of pregnancy (ICP), and benign recurrent intrahepatic cholestasis^13^. Infections, autoimmune, metabolic diseases and drug side effects often results in cholestasis^14^. Patients with cholestasis experience severe and intractable pruritus^15^,^16^, and also exhibit altered pain perception^17^,^18^. Analgesia has been observed in cholestatic patients, for example, patients undergoing orthotopic liver transplantation for cholestatic diseases reported lower postoperative pain scores and required less morphine for analgesia than patients undergoing liver resection^19^. It was demonstrated peripheral endogenous opioid-mediated and naloxone-reversible analgesia in mice or rats with bile duct ligation, which induced obstructive cholestatic disease^20^,^21^. Recently, there were several important findings to elucidate the pathogenesis of cholestatic pruritus. For example, serum concentrations of lysophosphatidic acid (LPA) and autotoxin (the enzyme that forms LPA) were significantly increased in cholestatic patients with pruritus and could directly activate dorsal root ganglia (DRG) neurons reflected by increased intracellular calcium^22^, suggesting LPA is a potential mediator of cholestatic pruritus. Bile acids, such as deoxycholic acid (DCA) and taurolithocholic acid (TLCA), bind to TGR5 (also known as GPR131 or GPBAR1) and activate transient receptor potential cation channel A1 (TRPA1) in primary sensory neurons and subsequently stimulates the release of itch-related neuropeptides, such as gastrin-releasing peptide (GRP) and natriuritic precursor peptide B (NPPB), in the spinal cord to transmit itch in mice^17^,^23^. Previous report also demonstrated that protease-activated receptor 2 (PAR2)-induced sensitization of transient receptor potential cation channel V1 (TRPV1) contributed to the pathogenesis of cholestatic pruritus in rats^24^. Although several novel itch mediators (LPA, bile acids and PAR2 agonists) and receptors (TGR5, TRPV1/A1, and PAR2) were demonstrated to be involved in cholestatic itch, the peripheral and central mechanisms underlying cholestatic pruritus and antinociception are still not completely understood.

Among monoamine neurotransmitters, serotonin, or 5-hydroxytryptamine (5-HT), is considered to play key and complex role in pain sensation^25^, and recently it was also demonstrated its crucial role in itch sensation^26^. To date, seven classes of 5-HT receptors (5-HT_1_-5-HT_7_) have been identified and comprise at least 15 subtypes^25^. Except 5-HT_3_ receptor as a ligand-gated cation channel, all other 5-HT receptors are G protein-coupled receptors (GPCRs)^25^. As 5-HT is not able to penetrate the blood-brain-barrier (BBB), peripheral and central 5-HT systems are considered to be separated compartments and employ distinct rate-limiting enzymes for 5-HT synthesis, such as tryptophan hydroxylase 1 (*Tph1*) in the skin and tryptophan hydroxylase 1 (*Tph2*) in the brain^26^. 5-HT in the skin released from mast cells, as a critical component of “inflammatory soup”, was identified as a potent inducer of pain or itch via activation distinct 5-HT receptors subtypes, such as 5-HT_2A_, 5-HT_2B_, 5-HT_2C_, 5-HT_3_, 5-HT_4_, 5-HT_6_, and 5-HT_7_^27^,^28^,^29^. Descending 5-HT pathways, originating from the nucleus raphe magus (NRM) within the vicinity of the rostral ventromedial medulla (RVM), may exert either inhibitory or facilitatory influence on pain or itch transmission in spinal cord dorsal horn, possible dependent on the physiological or pathophysiological status^26^,^30^,^31^. The discovery of multiple 5-HT receptors subtypes may explain their complex influence on the processing of pain or itch signal^25^,^28^,^30^,^32^,^33^. Interestingly, several clinical trials found that administration of 5-HT_3_ receptor antagonist ondansetron^34^,^35^,^36^,^37^,^38^ or selective serotonin reuptake inhibitor (SSRI) sertraline^39^,^40^ were able to alleviate cholestatic pruritus, providing important clinical evidence to support critical role of 5-HT system in cholestatic pruritus. Although the importance of 5-HT system is emphasized in the modulation of pain and itch^25^,^26^,^41^, the possible roles of peripheral and spinal 5-HT receptors in itch and antinociception induced by cholestasis are still unclear.

The aim of the present study was to reveal the possible roles of peripheral and spinal 5-HT receptors in itch and antinociception in common bile duct ligation (BDL) rats, a severe model of obstructive cholestasis. We firstly demonstrated behavioral phenotypes for itch and antinociception in BDL rats and then examined the changes of the 5-HT level in the skin and spinal cord. Furthermore, we used quantitative real-time polymerase chain reaction (qPCR) to screen the expression change of difference 5-HT receptors subtypes at the peripheral and central nervous systems in BDL rats. Finally, we employed several pharmacological 5-HT receptors agonists and antagonists to elucidate the distinct roles of 5-HT receptors subtypes, especially 5-HT_1A_, 5-HT_2_, 5-HT_3_ and 5-HT_7_, in the modulation of itch and antinociception in BDL rats.

## Materials and Methods

### Animals

Adult male Sprague-Dawley rats (weighing 200–250 g) were obtained from the Shanghai SLAC Laboratory Animal CO. LTD. Rats were housed in groups of 3 rats per cage with food and water available *ad libitum* and kept in controlled room temperature (22 ± 2 °C) and humidity (60–80%) under a 12 h/12 h light/dark cycle. All experimental procedures and animal handing were performed in accordance with the guidelines of the International Association for the Study of Pain and the animal protocols were approved by Soochow University Animal Committee. The authors tried all efforts to minimize the number of animals used.

### Surgical laparotomy and common bile duct ligation (BDL)

The surgical laparotomy and common bile duct ligation was performed as previously described^42^,^43^. Briefly, laparotomy was performed under anesthesia with isoflurane and common bile duct was ligated using two ligatures. Rats with laparotomy, bile duct identification without ligation were used as sham rats. The body temperature of rat was maintained at 37 ± 1 °C during the entire surgical procedure. Cholestasis was confirmed by increased serum level of bilirubin as well as the intact ligature and proximal dilation of the common bile duct at the sacrifice time.

### Drugs and administration

Serotonin hydrochloride (5-HT), deoxycholic acid (DCA), 5-HT_2_ receptor agonist α-methylserotonin maleate salt (α-methyl-5-HT), 5-HT_3A_ receptor agonist 2-Methyl-5-hydroxytryptamine hydrochloride (2-Methyl-5-HT), and the 5-HT_2A_ receptor antagonist ketanserin tartrate salt (ketanserin) was obtained from Sigma-Aldrich (St. Louis, MO, USA). The 5-HT_1A_ receptor agonist 8-hydroxy-2-(dipropylamino) tetralin hydrobromide (DPAT), the 5-HT_1A_ receptor antagonist WAY-100635 maleate salt, the 5-HT_3A_ receptor agonist 2-Methyl-5-hydroxytryptamine hydrochloride (2-Methyl-5-HT), 5-HT_7_ receptor antagonist (2R)-1-[(3-Hydroxyphenyl)sulfonyl]-2-[2-(4-methyl-1-piperidinyl)ethyl]pyrrolidine hydrochloride (SB269970) were obtained from Tocris Bioscience (Bristol, UK). 5-HT_3A_ receptor antagonist Ondansetron Hydrochloride Injection was obtained from China Jiangsu yabang Pharmaceytical factory CO. Ltd. (Jiangsu, China). The 5-HT re-uptake inhibitor Fluoxetine Hydrochloride Dispersible Tablets (PROZAC) was obtained from Patheon France. Ketanserin, WAY-100635 and Fluoxetine were dissolved in 10% DMSO and other reagents were dissolved in sterile saline if not specified. The information of 5-HT receptor agonists and antagonists used in this study was list in Table 1.

After BDL surgery 5, 10, 15, and 30 days, intradermal injection of 5-HT (200 μg) into the rat cheek (total volume 20 μl) or neck (total volume 50 μl) was perform to assess itch response in the sham and BDL rats. After BDL surgery 15 days, intradermal injection 5-HT receptors agonists into the rat cheek (total volume 20 μl), including 5-HT_1A_ receptor agonist 8-hydroxy-2-(dipropylamino) tetralin hydrobromide (DPAT; 100 μg), 5-HT_2A_ receptor agonist α-methylserotonin maleate salt (α-methyl-5-HT; 100 μg), 5-HT_3_ receptor agonist 2-Methyl-5-hydroxytryptamine hydrochloride (2-Methyl-5-HT; 100 μg), 5-HT_7_ receptor agonist 4-[2-(Methylthio)phenyl]-N-(1,2,3,4-tetrahydro-1-naphthalenyl)-1-piperazinehexanamide hydrochloride (LP44; 100 μg) was perform to assess itch response in the sham and BDL rats. After BDL surgery 15 days, we also examined the effects of intrathecal injection of 5-HT receptors agonists on itch and antinociception in the sham and BDL rats. The doses used were as follow: DPAT (10 μg), α-methyl-5-HT (10 μg), 2-Methyl-5-HT (10 μg), and LP44 (10 μg). After BDL surgery 15 days, the effects of 5-HT receptors antagonists on itch and antinociception in the sham and BDL rats were assessed by intrathecal (i.t.) or intraperitoneal (i.p.) injection of them, including 5-HT_1A_ receptor antagonist WAY-100635 maleate salt (i.t. 10 μg), 5-HT_2A_ receptor antagonist ketanserin tartrate salt (i.p. 1 mg/kg), 5-HT_3_ receptor antagonist ondansetron hydrochloride (i.p. 3 mg/kg), and 5-HT_7_ receptor antagonist (2R)-1-[(3-Hydroxyphenyl)sulfonyl]-2-[2-(4-methyl-1-piperidinyl)ethyl]pyrrolidine hydrochloride (SB269970; i.t. 10 μg). The injection of 5-HT receptors agonists or antagonists was performed before 30 min of i.d. injection of 5-HT (200 μg) and the doses used in this study were based on previous reports^29^,^59^,^60^,^61^,^62^ and our pilot experiments.

### Neck model of itch

As described previously^12^,^59^,^63^, rats were shaved at the nape of the neck at least 2 day before experiments. On the day of behavioral testing, rats were individually placed in small plastic chambers (15 × 20 × 25 cm) on an elevated metal mesh floor and allowed at least 60 min for habituation. Under brief anesthesia of isoflurane, rats were given an intradermal injection of 50 μl of 5-HT (200 μg) via a 26 G needle into the nape of the neck. Immediately after the injection, rats were returned to their chambers and were video recorded for 60 min. The video was subsequently played back offline and itch behavior was quantified by counting the number of scratches in a blinded manner. A scratch was counted when a rat lifted its hind paw to scratch the shaved region and returned the paw to the floor or to the mouth for licking. The neck model of itch is usually used to test the possible effects of intrathecal or systemic injection of drugs.

### Cheek model of itch

As described previously^64^,^65^,^66^, we intradermally injected chemicals into the cheek of rats. Rats were shaved on cheeks (approx. 5 × 8 mm area) at least 2 day before the experiment. On the day of experiment, rats were intradermally injected of 20 μl of 5-HT (200 μg) via a 26G needle into the cheek under brief anesthesia with isoflurane. After the injection, rats were immediately returned to their chambers and were video recorded for 60 min. The video was played back and the wipes and scratches were quantified by counting their number in a blinded manner. Scratching is counted when rats scratch the injection site on the cheek by hind paw and then returning to the floor or to the mouth. Wiping is counted when rats unilaterally wipe the injected site using the forelimb, which was not part of grooming behavior. As intrathecal injection of drugs by lumbar puncture may be difficult to reach the central nerve system that innervates the cheek area, the cheek model of itch is usually used to test the possible effects of systemic injection of drugs.

### Hargreaves test

According to previously report^67^, we put the rats in plastic boxes and measured the hindpaw withdrawal latency to radiant heat apparatus (IITC Life Science). Rats were placed on a glass floor maintained at 25 °C in a clear plastic chamber and a radiant heat source, which was located under the glass floor, was focused onto the plantar surface of the hindpaw. We measured paw withdrawal latency 2–3 times for each hindpaw, and we used the mean of the results for analysis. We set a cut off of 20 s to prevent potential tissue injury.

### Tail immersion test

As previously described^43^,^66^, tail immersion test was employed to determine heat pain sensitivity in rats. Briefly, the terminal 3 cm of a rat’s tail was immersed in hot water bath at 52 °C and the latency of tail flick was recorded with a cutoff time of 15 seconds to avoid tissue damage.

### von Frey filament test

To test mechanical hypersensitivity, we determined the mechanical sensitivity of hind paws by using series of von Frey filaments (4 g, 15 g, and 26 g) as previous report^66^. Rats were placed on a metal mesh floor and von Frey filaments were applied from underneath the floor. The plantar surface of hind paw was received consecutively by 10 stimulations. The measurement was repeated twice at an interval of 10 minutes. We used response frequency to estimate the mechanical sensitivity of rats.

### Rota-rod test

We used Rota-rod test to evaluate the motor function of rats at day 15 after BDL surgery^43^,^68^. Before testing 2 days, all of the rats were trained at various speeds of rotation. Each rat was placed on the rungs and allowed to remain stationary at 0 rpm for 10 sec, after which the speed was increased to 5 rpm for a maximum of 5 min. Rats were tested at 5, 10 until 25 rpm, during this time, rats that fell off the cylinder were placed back on the rotarod for three times. All rats were tested at 25 rpm for the 5-min period for each animal. Rats were tested for three trials with an interval of 20 min and the latency for falling was averaged.

### RNA isolation and quantitative real-time polymerase chain reaction (qPCR)

The sham and BDL rats were sacrificed at 15 days after surgery. The rat brainstem, trigeminal ganglia, cervical DRGs and spinal cord were dissected out and collected. Total RNA was extracted by homogenizing tissues using Trizol Reagent (Invitrogen, Carlsbad, California) according to the protocol supplied by manufacturer. Chloroform (Sigma-Aldrich) was added after homogenization and the tubes were vortexed, followed by incubation at room temperature for 5 min and centrifugation at 14,000 rpm for 20 min at 4 °C. The supernatant was transferred to a new tube and isopropanol was added. The aqueous phase was centrifuged at 14,000 RPM for 20 min at 4 °C. Pellets were washed using 70% ethanol and resuspended in diethylpyrocarbonate (DEPC)-treated water. The purity and concentration of RNA were determined with NanoDrop ND-1000 Spectrophotometer (NanoDrop Technologies, Wilmington, DE, USA) with absorbance at 260 and 280 nm. One microgram of total RNA was reverse transcribed for synthesizing cDNA using RevertAid First Strand cDNA Synthesis Kit, according to the protocol supplied by the manufacturer (Thermo Fisher Scientific, Waltham, USA). qPCR experiment was performed by SYBR Green PCR Master Mix (Roche, Basle, Switzerland) using Opticon real-time PCR Detection System (ABI 7500, Life technology, USA). Data were normalized to the housekeeping gene β-actin. The 2(-Delta Delta C(t)) method was used to analyze the relative level of gene expression. Primers used are listed in Table 2.

### Immunofluorescence

Rats were deeply anesthetized with chloral hydrate and intracardially perfused first with saline, then with 4% of the fixative solution paraformaldehyde in 0.1 M phosphate buffer (pH = 7.4). The L4-6 lumbar spinal cords were collected and postfixed in the same solution overnight. The spinal cords were transferred into a phosphate-buffered 15% sucrose solution overnight. After that, they were transferred into a phosphate-buffered 30% sucrose solution overnight. The spinal cord sections were cut at the thickness of 14-μm in a cryostat. The sections were mounted on siliconized slides for immunostaining. Sections were rinsed three times for 10 min with PBS, and then all incubated in PBS containing 10% normal goat serum (GIBCO) and 0.3% Triton X-100 (Sigma) for 1 h. Then, the sections were incubated overnight at 4 °C in rabbit polyclonal anti-5-HT antibody (diluted 1:500; Sigma, S5545, USA). We used the same anti-5-HT antibody as Prof. Yaobo Liu’s laboratory and the specificity of the antibody had been demonstrated by his lab^69^. After this, the sections were rinsed three times for 10 min with PBS and then incubated in a solution containing a goat anti-rabbit secondary antibody conjugated to Alexa Fluor 488 (1:500; Molecular Probes) for 1 h. They were then rinsed three times for 10 min with PBS, quickly rinsed with ddH_2_O, and left to dry on a plate at 37 °C for 15 min. Finally, the slice sections were mounted on glass slides and coverslipped with a drop of mounting medium (Dako North America Inc., Carpinteria, CA, USA). The coverslip was sealed with nail polish for preventing drying and movement. All slice sections were stored in dark at 4 °C. The sections were observed and photographed were examined under a Zeiss fluorescence microscope AXIO SCOPE A1 (Oberkochen, Germany), and images were analyzed with AxioVision software.

### Histology

Rats were terminally anesthetized with isoflurane and transcardially perfused with phosphate buffersaline (pH 7.4, 0.1 M) followed by fixation with 4% (w/v) paraformaldehyde. Liver samples were post-fixed in 4% (w/v) formalin prepared in PBS for 24 h. This was followed by the dehydration of fixed tissue in various grades of alcohol (70%, 90%, 100%, v/v) and then cleared in benzene. Samples were removed; transverse sections were embedded in molten paraffin wax and 5 μm thick sections were cut using a microtome. Liver sections were stained with standard H&E staining. The images were analyzed using Adobe PhotoShop and the average number of bile duct-like structures per high-power field (HPF) was quantified.

### High performance liquid chromatography (HPLC) analysis for 5-HT

Rats were terminally anesthetized with isoflurane on day 15 after BDL surgery. The skin and lumbar spinal cords were dissected quickly, weighed and then homogenized with an ultrasonic homogenizer (Microsonic, Dortmund, Germany) in 400 μl 0.4 mol/L perchloric acid. Samples were then centrifuged at 15000 rpm at 4 °C for 10 min, filtered through the 0.22 μm syringe filter and stored at −80 °C until HPLC analysis. The concentration of 5-HT were measured by applying reverse-phase HPLC with electrochemical detection (Waters, USA). The reversed phase column was YWG-C_18,_ which was perfused for analysis with amobile phase composed of 0.1 mol/L NaAc (including 0.1 mol/L EDTA-Na_2_) and 10% methanol at pH 5.1. The flow rate was 1 mL/min. The data was quantified using the area under the peaks and external standards. The obtained results were presented in ng per gram of wet tissue (ng/g).

### Statistical analysis

All data were analyzed using GraphPad Prism 6 software (GraphPad, San Diego, CA, USA). All data were expressed as the mean ± S.E.M. The statistical significance between two groups was analyzed by unpaired two-tailed Student’s *t*-test. One-way ANOVA followed by post-hoc Bonferroni test was used for multiple comparisons. Two-way repeated-measured ANOVA was also used to analyze the data with multiple time points. Differences with *P* < 0.05 were considered as statistical significance.

## Results

### Obstructive cholestasis model was established by common bile duct ligation (BDL) in rats

**Figure 1:**
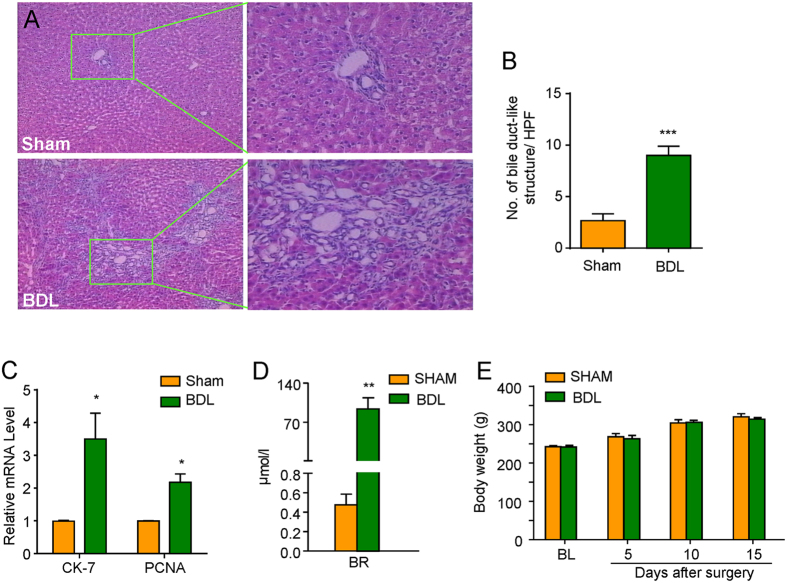
Cholestasis was induced by common bile duct ligation (BDL) in rats. (**A**) Representative H&E staining photomicrographs of liver section in sham and BDL rats. Compared to SHAM control rats, the bile duct had obvious atypical hyperplasia in the BDL rats at the 4^th^ week after surgery (×200). The right pictures are higher magnification of boxed area in left pictures. (**B**) Quantification of bile duct-like structure in liver sections from the sham and BDL rats. (**C**) qPCR analysis showed that the mRNA expression of CK-7 and proliferating cell nuclear antigen (PCNA) were significantly increased in BDL rats compared to the sham rats. (**D**) The level of serum total bilirubin (BR) was significantly increased in BDL rats compared to sham rats at the 4^th^ week after BDL surgery. *n* = 4–6 per group. (**E**) There was no significant difference for the body weight between sham and BDL rats (*n* = 12 per group) (^**^P < 0.01 versus sham group and analyzed by Student’s *t*-test or two-way ANOVA followed by Bonferroni’s test).

We firstly established an obstructive cholestatic rat model by common bile duct ligation (BDL). The biochemical and morphological evidence of liver injury and cholestasis were confirmed in BDL rats by multiple approaches (Fig. 1). H&E staining of liver section in BDL rats showed typical features of cholestasis, including expansion of the liver capillary bile duct, fibrosis around the small bile duct, and connection of the portal area (Fig. 1A). The bile duct-like structures in liver sections from BDL rats were increased compared to the sham rats (Fig. 1B). In addition, qPCR analysis showed that the mRNA expression of CK-7, which is a specific cholangiocytes marker, and proliferating cell nuclear antigen (PCNA), which is a cell proliferation-related marker, were significantly increased in BDL rats compared to the sham rats (Fig. 1C). Thus, the results showed that the bile duct had obvious hyperplasia in the BDL rats at the 4^th^ week after surgery. The increased serum total bilirubin (BR) was also demonstrated in BDL rats (Fig. 1D; t_8_ = 2.508; *P* = 0.0365). In accordance with previous study^70^,^71^, There was no significant difference for the body weight between the sham and BDL rats within 1 to 15 days after BDL surgery (Fig. 1E; F_(1,52)_ = 0.1752; *P* = 0.6772).

### 5-HT-evoked itch behavior was significantly enhanced in BDL rats

**Figure 2:**
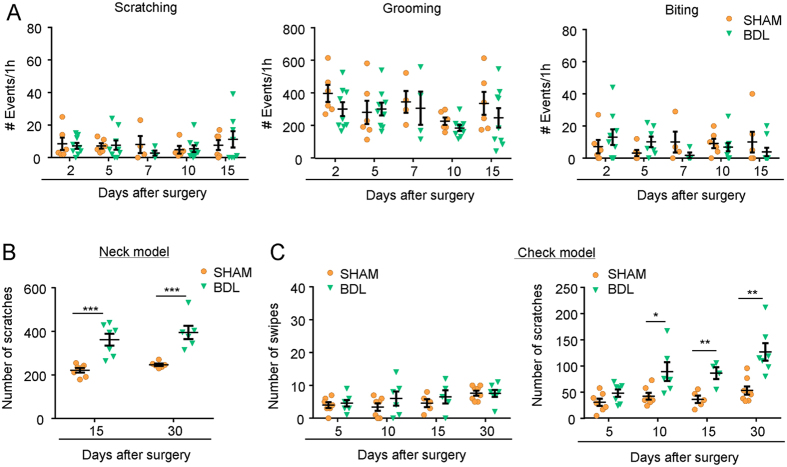
5-HT-induced itch was enhanced in BDL rats compared to sham control. (**A**) Spontaneous itch-related behaviors, such as scratching, grooming, and biting, were not changed in BDL rats compared to sham group (*n* = 6 per group). (**B**) The scratching behavior induced by i.d. injection of 5-HT (200 μg) into the nape of the neck of rats was significantly increased in BDL rats compared to sham group (*n* = 6 per group). (**C**) The scratching behavior, but not wiping behavior, induced by i.d. injection of 5-HT (200 μg) into the cheek of rats was significantly increased in BDL rats compared to sham group (*n* = 6 per group) (^*^P < 0.05, ^**^P < 0.01, ^***^P < 0.001 versus sham values and analyzed by two-way ANOVA followed by Bonferroni’s test).

Previous report by Belghiti M *et al*. demonstrated spontaneous scratching behavior was significantly increased in BDL rats^24^, suggesting BDL rat may be a suitable model for exploring the mechanisms underlying cholestatic itch. Aimed to replicate this cholestatic itch model in rats, we carefully observed and quantified the spontaneous behaviors after BDL surgery, such as scratching, grooming, and biting behaviors, which may reflect chronic itch in rats^24^. Surprisingly, none of aforementioned itch-related behaviors showed significant difference between the sham and BDL rats (Fig. 2A). This discrepancy may be attributed to the different rat stains employed: we used SD rats while Belghiti M *et al*. used Wistar rats. Our results argued lack of BDL-associated spontaneous itch in SD rats. To further determine whether chemical-evoked itch sensation was changed in BDL rats, we next investigated whether 5-HT-evoked itch was affected in BDL rats. After BDL surgery 15 to 30 days, scratching induced by intradermal (i.d.) injection of 5-HT into the nape of the neck was significant enhanced (Fig. 2B; F_(1,22)_ = 45.76; *P* < 0.0001) in BDL rats. In order to distinguish itch and pain behaviors in rats, we used the cheek model by i.d. injection of chemicals into cheek of rats, which demonstrated that painful stimuli elicit forelimb wiping, while itchy stimuli elicit hindlimb scratching^72^. After BDL surgery 10 to 30 days, scratching induced by i.d. injection of 5-HT into the cheek was significant enhanced (Fig. 2C; F_(1,43)_ = 34.88; *P* < 0.0001) in BDL rats. In contrast, there was no significant difference for the wiping behavior induced by 5-HT in cheek model between the sham and BDL rats (Fig. 2C; F_(1,43)_ = 1.775, *P* = 0.1898).

### Antinociception responding to mechanical and heat stimuli was induced in BDL rats

**Figure 3:**
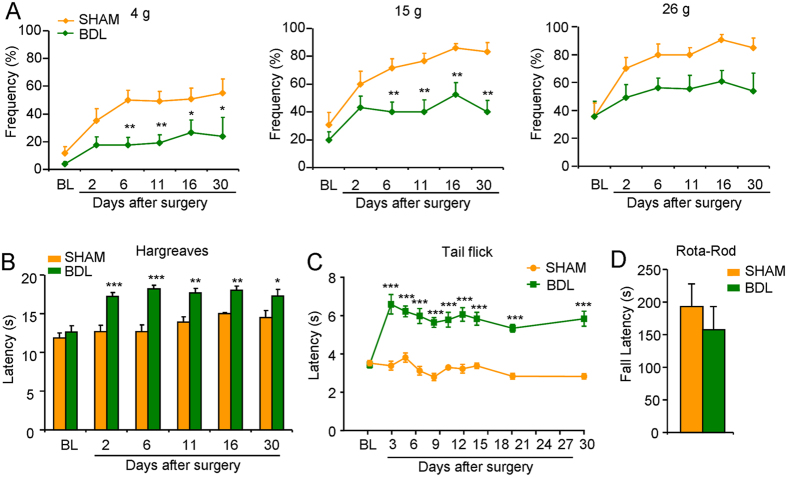
Antinociception responding to heat and mechanical stimuli was induced in BDL rats. (**A**) We used von Frey filament (4 g, 15 g and 26 g) to evaluate mechanical sensitivity in sham and BDL rats. Compared to sham rats, BDL rats showed reduced mechanical sensitivity 6 to 30 days after BDL surgery (*n* = 8–12 per group). (**B**) The Hargreaves test showed that paw withdrawal thermal latency was significantly increased in the BDL rats compared to sham control 6 to 30 days after BDL surgery (*n* = 6–8 per group). (**C**) The tail-immersion test showed that latency of tail-flick in response to 52 °C hot water was significantly increased in the BDL rats compared to sham group 3 to 30 days after BDL surgery (*n* = 8 per group). (**D**) There was no significant difference for the duration to fall from the rod between sham and BDL rats (*n* = 8 per group) (^*^P < 0.05, ^**^P < 0.01, ^***^P < 0.001 versus sham values and analyzed by Student’s *t*-test or two-way ANOVA followed by Bonferroni’s test).

We next asked whether pain sensation was affected in BDL rats. After BDL surgery 6 to 30 days, the mechanical sensitivity, evaluated by von Frey test, was significantly lower in BDL rats than that in sham rats (Fig. 3A; 4g: F_(1,125)_ = 39.40, *P* <  0.0001; 15 g: F_(1,125)_ = 44.21, *P* < 0.0001; 26 g: F_(1,126)_ = 19.52, *P* < 0.0001). The heat sensitivity was determined by Hargreaves test and tail-flick test. The results showed the paw thermal withdrawal latency to radiated heat was significantly increased from 2 to 30 days following BDL surgery in rats (Fig. 3B; F _(1,104)_ = 60.80, *P* < 0.0001). Tail-flick latency to 52 °C hot water was also significantly prolonged in BDL rats (Fig. 3C; F _(1,200)_ = 350.2, P < 0.0001). After BDL 30 days, there was no significant difference for the falling latency evaluated by Rota-rod test between sham and BDL rats (Fig. 3D), suggesting that pain or itch phenotypes in BDL rats may not be attributed to the motor coordination dysfunction.

### The levels of 5-HT in the skin and spinal cord were increased in BDL rats

**Figure 4:**
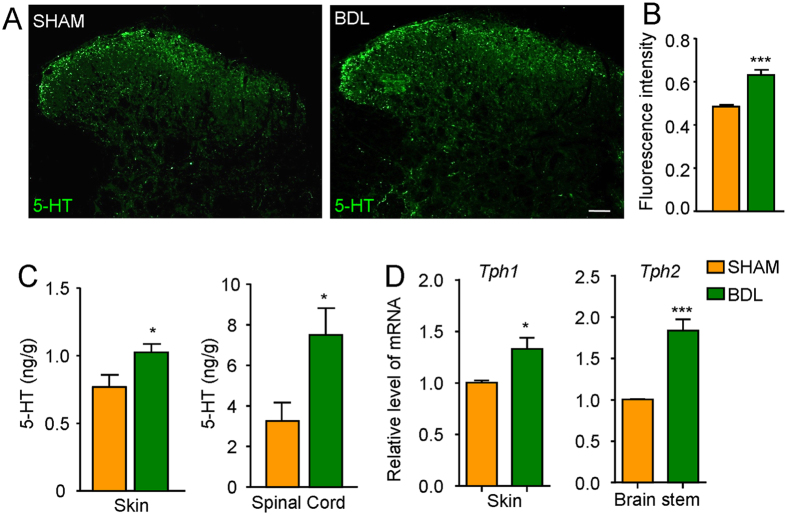
The 5-HT level in the skin and the spinal cord significantly increased in BDL rats. (**A**) Representative photomicrographs showing the immunostaining of 5-HT in spinal cord dorsal horn of the sham and BDL rats 15 days after BDL surgery. Scale bar: 100 μm. (**B**) The quantitative analysis showed that the 5-HT-positive immunofluoresence density significantly increased in spinal cord BDL rats compared to sham rats 15 days after BDL surgery (*n* = 4 per group). (**C**) HPLC analysis showed that 5-HT level in the skin and the spinal cord significantly increased in BDL rats compared to sham rats 15 days after BDL surgery (*n* = 5 per group). (**D**) qPCR analysis showed the mRNA expression of tryptophan hydroxylase 1 (*Tph1*) in the skin and tryptophan hydroxylase 1 (*Tph2*) in the brain stem, the rate-limiting enzymes for 5-HT synthesis, were up-regulated in BDL rats compared to sham rats. (^*^P < 0.05, ^***^P < 0.001 versus sham values and analyzed by Student’s *t*-test).

We subsequently evaluated the changes of 5-HT level in the skin and the lumbar spinal cord in sham and BDL rats by using immunofluorescence and high-performance liquid chromatography (HPLC) analysis. The immunostaning of 5-HT in the spinal cord dorsal horn was increased in BDL rats compared to sham rats (Fig. 4A,B; t_14_ = 5.707, *P* < 0.0001). As shown in Fig. 4, HPLC analysis also confirmed that 5-HT level in the skin (t_7_ = 2.435, *P* = 0.0451) and the spinal cord (t_6_ = 2.594, *P* = 0.0410) significantly increased in BDL rats (Fig. 4C). To determine the origin of increased 5-HT levels in the skin and spinal cord, we further used qPCR to examine the mRNA expression of tryptophan hydroxylase 1 (*Tph1*) in the skin and tryptophan hydroxylase 1 (*Tph2*) in the brain stem, the rate-limiting enzymes for 5-HT synthesis^26^. The results showed that the expression of both *Tph1* in the skin and *Tph2* in the brain stem were up-regulated in BDL rats (Fig. 4D). Thus, the results suggested that increased 5-HT level in the skin and the spinal cord, possible due to the up-regulated 5-HT synthesis, may actively participate in itch and antinociception under cholestasis condition.

### The expression profile of 5-HT receptor subtypes changed in peripheral and central nervous system (CNS)

**Figure 5:**
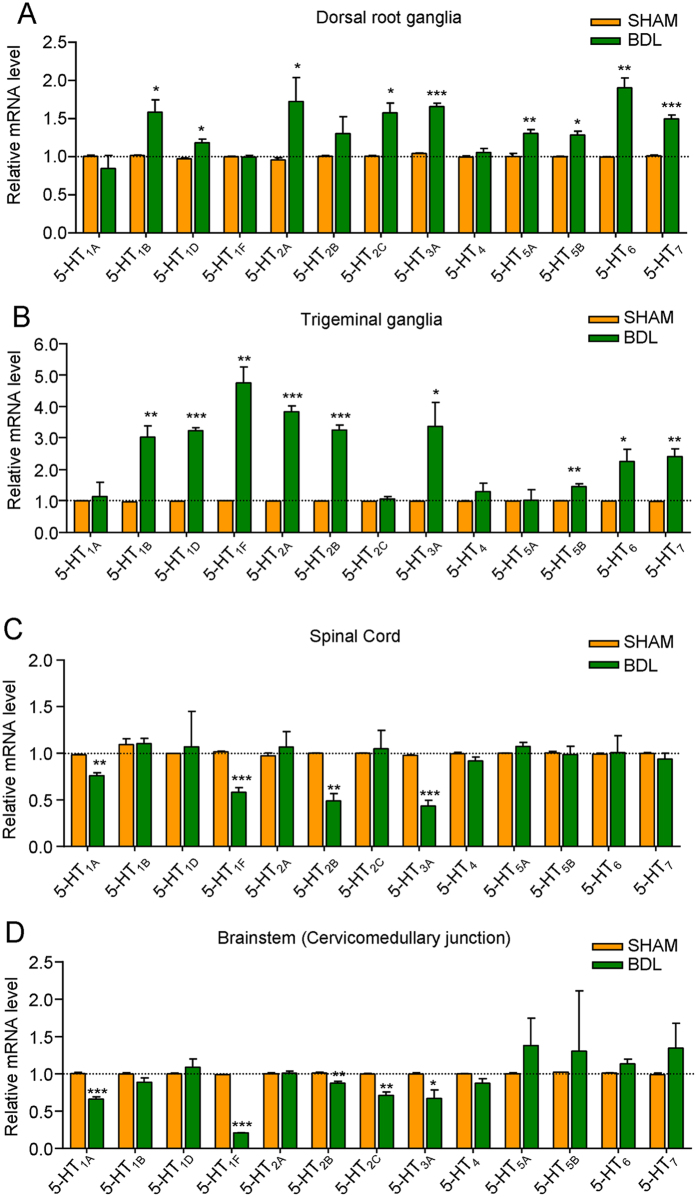
Q-PCR analysis revealed the expression profile of 5-HT receptor subtypes in peripheral and central nervous system (CNS) in sham and BDL rats. Real-time quantitative PCR (qPCR) analysis showed that mRNA expression changes of different 5-HT receptors subtypes in dorsal root ganglia (**A**), trigeminal ganglia (**B**), spinal cord (**C**), and brainstem (**D**). Notably, multiple 5-HT receptors were up-regulated in dorsal root ganglia or trigeminal ganglia from BDL rats, including 5-HT_1B_, 5-HT_1D_, 5-HT_1F_, 5-HT_2A_, 5-HT_2C_, 5-HT_3A_, 5-HT_5A_, 5-HT_5B_, 5-HT_6_, and 5-HT_7_. In contrast, several 5-HT receptors were down-regulated in the spinal cord or brainstem from BDL rats, including 5-HT_1A_, 5-HT_1F_, 5-HT_2B_, 5-HT_2C_ and 5-HT_3A_. (*n* = 6 per group) (^*^P < 0.05; ^**^P < 0.01, ^***^P < 0.001 versus sham values and analyzed by Student’s *t*-test).

We next used qPCR to examine the expression profile changes of 5-HT receptor subtypes in peripheral and central nervous system (especially spinal cord and brain stem). The results demonstrated that mRNA expression of multiple 5-HT receptors, including 5-HT_1B_, 5-HT_1D_, 5-HT_2A_, 5-HT_2C_, 5-HT_3A_, 5-HT_5A_, 5-HT_5B_, 5-HT_6_, and 5-HT_7_, were up-regulated in dorsal root ganglia (DRG) from BDL rats compared to sham rats (Fig. 5A). The results also demonstrated that mRNA expression of multiple 5-HT receptors, including 5-HT_1B_, 5-HT_1D_, 5-HT_1F_, 5-HT_2A_, 5-HT_2B_, 5-HT_3A_, 5-HT_5B_, 5-HT_6_, and 5-HT_7_, were up-regulated in trigeminal ganglia (TG) from BDL rats compared to sham rats (Fig. 5B). In sharp contrast, the mRNA expression of multiple 5-HT receptors, including 5-HT_1A_, 5-HT_1F_, 5-HT_2B_, and 5-HT_3A_, were down-regulated in the spinal cord from BDL rats compared to sham rats (Fig. 5C). Similarly, the mRNA expression of multiple 5-HT receptors, including 5-HT_1A_, 5-HT_1F_, 5-HT_2B_, 5-HT_2C_, and 5-HT_3A_, were down-regulated in the brain stem (especially cervicomedullary junction) from BDL rats compared to sham rats (Fig. 5D). Thus, these results suggested up-regulation of 5-HT receptors in primary sensory neurons in DRG or TG and down-regulation of 5-HT receptors in the spinal cord or brainstem may contribute to the enhanced itch behavior and antinociception in BDL rats. We subsequently performed pharmacological experiments to investigate the possible roles of four 5-HT receptors subtypes, including 5-HT_1A_, 5-HT_2_, 5-HT_3_ and 5-HT_7_, in itch and antinociception in BDL rats.

### Peripheral 5-HT receptors, especially 5-HT_2_, 5-HT_3_ and 5-HT_7,_ contributed to enhanced itch response in BDL rats

**Figure 6:**
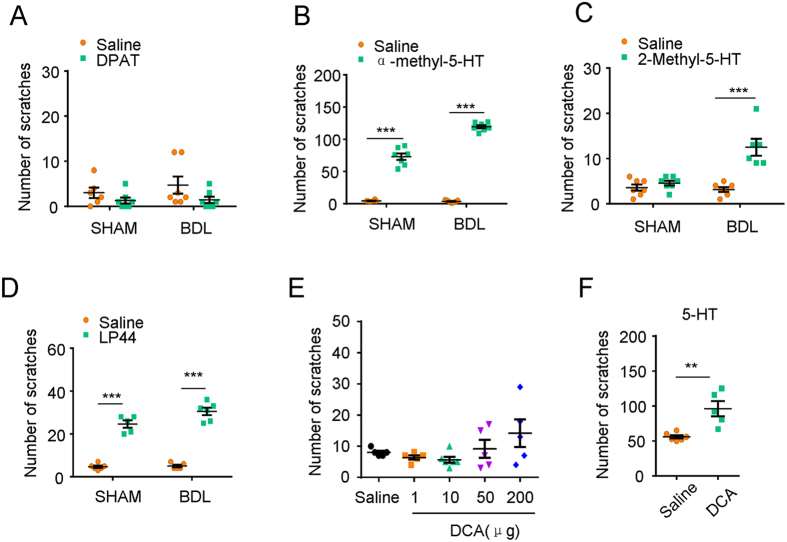
The effects of intradermal injection of 5-HT receptor agonists in sham and BDL rats. (**A–D**) The scratching response was induced by intradermal (i.d.) injection of 5-HT_1A_ receptor agonist DPAT (**A**) 100 μg, 5-HT_2_ receptor agonist α-methyl-5-HT (**B**) 100 μg, 5-HT_3_ receptor agonist 2-Methyl-5-HT (**C**) 100 μg, 5-HT_7_ receptor agonist LP44 (**D**) 100 μg into cheek of sham and BDL rats 15 days after BDL surgery (*n* = 6–8 per group). (**E**) No obvious scratching behavior induced by i.d. injection of DCA (1 to 200 μg into the cheek of naïve rats (*n* = 6–8 per group). (**F**) 15 minutes after i.d. injection of DCA (50 μg), the rats were given an i.d. injection 5-HT (20 μg) into the cheek. 5-HT-induced scratching behavior was significantly enhanced by DCA in naïve rats (*n* = 6–8 per group) (^**^P < 0.01, ^***^P < 0.001 vs. vehicle values and analyzed by Student’s *t*-test or one-way AVOVA following Bonferroni post hoc test).

We tried to identify which subtypes of 5-HT receptors in the periphery are involved in the enhanced itch behavior in BDL rats through i.d. injection of 5-HT receptors agonists into rat cheek. We found that i.d. injection of 5-HT_1A_ receptor agonist 8-hydroxy-2-(dipropylamino) tetralin hydrobromide (DPAT; 100 μg) failed to induce scratching behavior in both sham and BDL rats (Fig. 6A). In contrast, i.d. injection of 5-HT_2_ receptor agonist α-methylserotonin maleate salt (α-methyl-5-HT; 100 μg) was able to induce scratching behavior in both sham and BDL rats (Fig. 6B; for sham: t_11_ = 12.43; *P* < 0.0001; for BDL: t_12_ = 46.09; *P* < 0.0001). Interestingly, i.d. injection of 5-HT_3_ receptor agonist 2-Methyl-5-hydroxytryptamine hydrochloride (2-Methyl-5-HT; 100 μg) could only induce scratching response in BDL rats, but not in sham rats (Fig. 6C; for sham: t_12_ = 1.156; *P* = 0.2702; for BDL: t_11_ = 5.216; *P* = 0.0003). Finally, i.d. injection of 5-HT_7_ receptor agonist 4-[2-(Methylthio)phenyl]-N-(1,2,3,4-tetrahydro-1-naphthalenyl)-1-piperazinehexanamide hydrochloride (LP44; 100 μg) was also able to induce scratching behavior in both sham and BDL rats (Fig. 6D; for sham: t_9_ = 11.93; *P* < 0.0001; for BDL: t_8_ = 11.52; *P* < 0.0001).

As recent work showed that bile acid, such as deoxycholic acid (DCA) was able to induce itch behavior in mice via activation of TGR5 and TRPA1 in primary sensory neurons in DRG, suggesting bile acid may contribute to cholestatic itch^17^,^23^. We also tried to investigate the possible itch-inducing effect of DCA in rats and the interaction of peripheral bile acid and 5-HT system. Unexpected, i.d. injection of DCA (1 to 200 μg) into cheek failed to induce scratching behavior in rats (Fig. 6E). Thus, although bile acid can induce itch in mice^17^,^23^, our results clearly showed that bile acid was not able to induce scratching in rats, suggesting species difference for bile acid-induced itch. Interestingly, co-administration of DCA was able to enhance 5-HT-induced scratching in rats (Fig. 6E; t_9_ = 3.999; *P* = 0.0031), suggesting DCA may potentiate 5-HT-induced itch in rats.

### Increasing level of 5-HT in the central nervous system (CNS) inhibited itch and induced antinociception in sham and BDL rats

**Figure 7:**
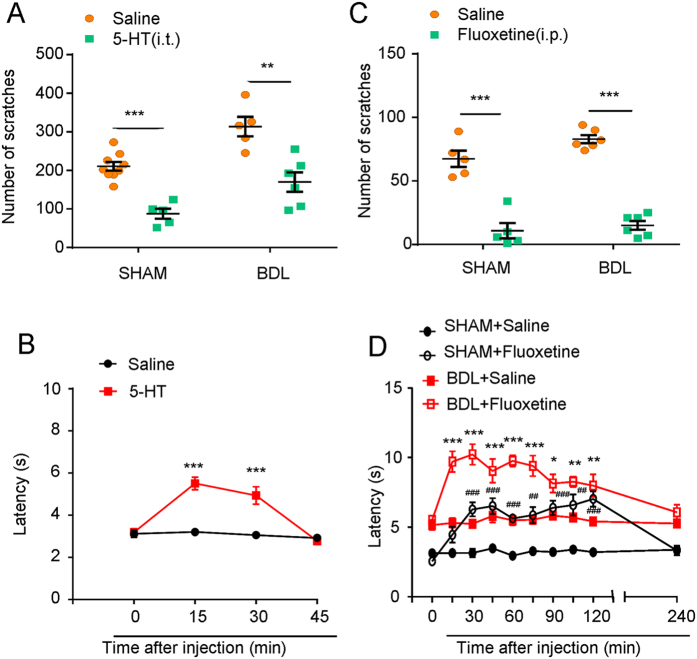
Enhanced 5-HT level in CNS inhibited itch and induced antinociception in sham and BDL rats. (**A**) Intrathecal (i.t.) injection of 5-HT (1 μg) significantly suppressed scratching induced by i.d. injection of 5-HT (200 μg) into the nape of the neck in sham and BDL rats (*n* = 5–9 per group). (**B**) I.t. injection of 5-HT (1 μg) increased the latency of tail-flick response to 52 °C hot water in naïve rats (*n* = 6–8 per group). (**C**) Intraperitoneal (i.p.) injection of 5-HT reuptake inhibitor fluoxetine (10 mg/kg) significantly suppressed scratching induced by i.d. injection of 5-HT (200 μg) into the cheek of sham and BDL rats (*n* = 6–8 for each group) (^**^P < 0.01, ^***^P < 0.001 vs. saline values and analyzed by Student’s *t*-test or two-way AVOVA following Bonferroni post hoc test). (**D**) I.p. injection of 5-HT reuptake inhibitor fluoxetine (10 mg/kg) significantly increased the latency of tail-flick response to 52 °C hot water in sham and BDL rats in a time-dependent manner (*n* = 6–8 per group) (^*^P < 0.05; ^**^P < 0.01, ^***^P < 0.001 vs. saline values from sham rats; ^##^*P* < 0.01, ^###^*P* < 0.001 vs. saline values from BDL rats and analyzed by two-way AVOVA following Bonferroni post hoc test).

After we demonstrated the role of peripheral 5-HT and 5-HT receptors in itch, we subsequently investigate the role of 5-HT in the CNS for modulating itch and antinociception in sham and BDL rats. Firstly, we found that intrathecal (i.t.) injection of 5-HT (1 μg) could significantly inhibit i.d. injection of 5-HT (200 μg)-induced scratching in both sham and BDL rats (Fig. 7A; for sham: t_12_ = 6.854; *P* < 0.0001; for BDL: t_9_ = 4.003; *P* = 0.0031). Additionally, i.t. injection of 5-HT could significantly increase the tail-flick latency response to 52 °C hot water in naïve rats (Fig. 7B; F_(1,48)_ = 41.96, *P* < 0.0001). We also found that intraperitoneal (i.p.) injection of 5-HT re-uptake inhibitor fluoxetine (10 mg/kg) could significantly suppressed 5-HT-induced scratching in sham and BDL rats (Fig. 7C; for sham: t_8_ = 6.442; *P* = 0.0002; for BDL: t_10_ = 14.62; *P* < 0.0001). Finally, i.p. injection of fluoxetine also significantly increased the tail-flick latency response to 52 °C hot water in sham and BDL rats (Fig. 7D; for sham: F_(1,80)_ = 163.6, *P* < 0.0001; for BDL: F_(1,100)_ = 167.2, *P* < 0.0001). Thus, increasing 5-HT level in CNS suppressed itch and induced antinociception in sham and BDL rats.

### Itch could be modulated by 5-HT receptors agonists or antagonists in sham and BDL rats

**Figure 8:**
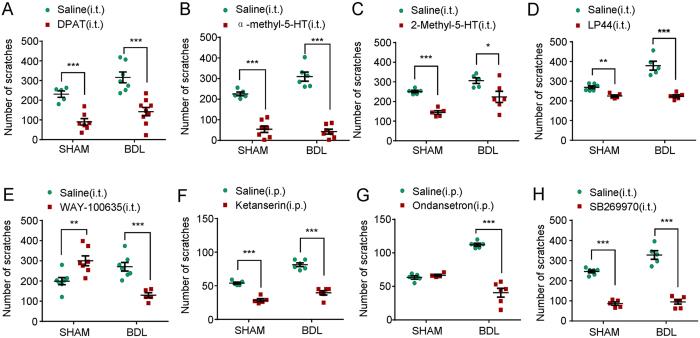
The effects of 5-HT receptors agonists or antagonists on 5-HT-induced scratching behavior in sham and BDL rats. (**A–D**) Scratching behavior induced by i.d. injection of 5-HT (200 μg) into the nape of the neck was significantly suppressed by intrathecal (i.t.) injection of 5-HT_1A_ receptor agonist DPAT (**A**) 10 μg, 5-HT_2_ receptor agonist α-methyl-5-HT (**B**) 10 μg, 5-HT_3_ receptor agonist 2-Methyl-5-HT (**C**) 10 μg, 5-HT_7_ receptor agonist LP44 (**D**) 10 μg in sham and BDL rats (*n* = 6–8 per group). (**E**) Intrathecal (i.t.) injection of 5-HT_1A_ receptor antagonist WAY-100635 (10 μg) increased 5-HT-induced scratching in sham rats, but suppressed that in BDL rats (*n* = 6–8 per group). (**F**) Intraperitoneal (i.p.) injection of 5-HT_2_ receptor antagonists ketanserin (1 mg/kg) could suppress 5-HT-induced scratching in both sham and BDL rats (*n* = 6–8 per group). (**G**) Intraperitoneal (i.p.) injection of 5-HT_3_ receptor antagonists ondansetron (3 mg/kg) can suppress 5-HT-induced scratching in BDL rats, but not sham rats (*n* = 6–8 per group). (**H**) Intrathecal (i.t.) injection of 5-HT_7_ receptor antagonist SB269970 (10 μg) could suppress 5-HT-induced scratching in both sham and BDL rats (*n* = 6–8 per group) (^*^P < 0.05, ^**^P < 0.01, ^***^P < 0.001 vs. vehicle values and analyzed by Student’s *t*-test).

In order to reveal the distinct roles of 5-HT receptor subtypes on itch response in sham and BDL rats, 5-HT receptors agonists or antagonists were administrated after BDL surgery 15 days. It was found that i.t. injection of 5-HT_1A_ receptor agonist DPAT (Fig. 8A; for sham: t_10_ = 5.799; *P* = 0.0002; for BDL: t_14_ = 5.102; *P* = 0.0002), 5-HT_2_ receptor agonist α-methyl-5-HT (Fig. 8B; for sham: t_10_ = 8.398; *P* < 0.0001; for BDL: t_11_ = 11.18; *P* < 0.0001), 5-HT_3_ receptor agonist 2-Methyl-5-HT (Fig. 8C; for sham: t_8_ = 9.602; *P* < 0.0001; for BDL: t_9_ = 2.433; *P* = 0.00378), and 5-HT_7_ receptor agonist LP44 (Fig. 8D; for sham: t_9_ = 4.746; *P* = 0.0010; for BDL: t_8_ = 6.597; *P* = 0.0002), suppressed 5-HT-induced scratching in both sham and BDL rats. Thus, these results suggested over-activation of 5-HT receptors subtypes 5-HT_1A_, 5-HT_2_, 5-HT_3_, and 5-HT_7_ might suppress itch in sham and BDL rats.

We next assessed the effects of intrathecal (i.t.) or intraperitoneal (i.p.) injection of 5-HT receptors antagonists on itch in sham and BDL rats after BDL surgery 15 days. The results showed that i.t. injection of 5-HT_1A_ receptor antagonist WAY-100635 increased 5-HT-induced scratching in sham rats (Fig. 8E; t_12_ = 3.328; *P* = 0.0060), but suppressed that in BDL rats (Fig. 8E; t_10_ = 5.101; P = 0.0005). It was found that i.p. application of 5-HT_2_ receptor antagonist ketanserin significantly inhibited 5-HT-induced scratching in both sham and BDL rats (Fig. 8F; for sham: t_8_ = 9.300; *P* < 0.0001; for BDL: t_10_ = 9.926; *P* < 0.0001). Interestingly, i.p. application of 5-HT_3_ receptor antagonist ondansetron hydrochloride failed to inhibit 5-HT-induced itch in sham rats (Fig. 8G; t_7_ = 1.011; *P* = 0.3456, but significantly inhibited that in BDL rats (Fig. 8G; t_9_ = 9.498; *P* < 0.0001). Finally, i.t. injection of 5-HT_7_ receptor antagonist SB269970 significantly inhibited 5-HT-induced scratching in both sham and BDL rats (Fig. 8H; for sham: t_9_ = 15.33; *P* < 0.0001; for BDL: t_8_ =9.374; *P* < 0.0001). Thus, these data suggested antagonism of 5-HT receptor subtypes 5-HT_1A_, 5-HT_2_, 5-HT_3_, and 5-HT_7_, might suppress itch response under cholestasis condition. It also suggested antagonism of 5-HT_1A_ might increase 5-HT-induced itch, however, antagonism of 5-HT_2_ and 5-HT_7_ might suppress itch under physiological condition.

### Antinociception could be modulated by 5-HT receptors agonists or antagonists in sham and BDL rats

**Figure 9:**
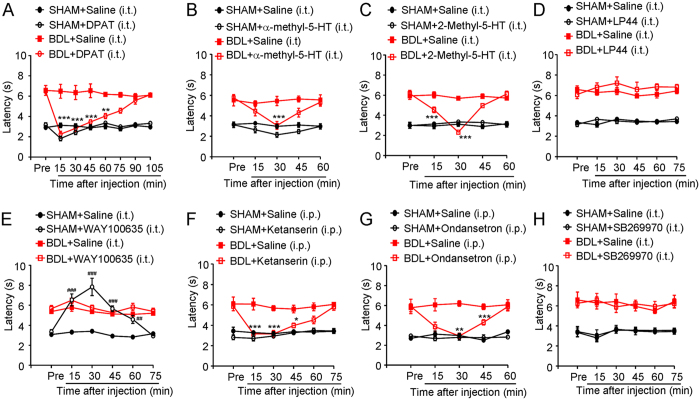
The effects of 5-HT receptors agonists or antagonists on antinociception in sham and BDL rats. (**A**) I.t. injection of 5-HT_1A_ receptor agonist DPAT decreased the latency of tail-flick response to 52 °C hot water in BDL rats, but not sham rats (*n* = 6–8 per group). (**B**) I.t. injection of 5-HT_2_ receptor agonist α-methyl-5-HT decreased the latency of tail-flick response to 52 °C hot water in sham and BDL rats (*n* = 6–8 per group). (**C**) I.t. injection of 5-HT_3_ receptor agonist 2-Methyl-5-HT decreased the latency of tail-flick response to 52 °C hot water in BDL rats, but not sham rats (*n* = 6–8 per group). (**D**) I.t. injection of 5-HT_7_ receptor agonist LP44 failed to change the latency of tail-flick response to 52 °C hot water in sham and BDL rats (*n* = 6–8 per group). (**E**) I.t. injection of 5-HT_1A_ receptor antagonist WAY-100635 significantly increased the latency of tail-flick in response to 52 °C hot water in sham and BDL rats (*n* = 6–8 per group). (**F**) I.p. application of 5-HT_2_ receptor antagonist ketanserin significantly reduced the latency of tail-flick in response to 52 °C hot water in BDL rats, but not sham rats (*n* = 6–8 per group). (**G**) I.p. application of 5-HT_3A_ receptor antagonist ondansetron significantly reduced the latency of tail-flick in response to 52 °C hot water in BDL rats, but not sham rats (*n* = 6–8 per group). (**H**) I.t. injection of 5-HT_7_ receptor antagonist SB269970 failed to change the latency of tail-flick in response to 52 °C hot water in sham and BDL rats (*n* = 6–8 per group) (^*^P < 0.05; ^**^P < 0.01, ^***^P < 0.001 vs. saline values from sham rats; ^##^*P* < 0.01, ^###^*P* < 0.001 vs. saline values from BDL rats and analyzed by two-way AVOVA following Bonferroni post hoc test).

In order to reveal the possible roles of 5-HT receptor subtypes on antinociception in sham and BDL rats, 5-HT receptors agonists or antagonists were administrated after BDL surgery 15 days. It was found that i.t. injection of 5-HT_1A_ receptor agonist DPAT (Fig. 9A; F_(1,72)_ = 82.89, *P* < 0.0001), 5-HT_2_ receptor agonist α-methyl-5-HT (Fig. 9B; F_(1,60)_ = 12.15, *P* = 0.0009), 5-HT_3_ receptor agonist 2-Methyl-5-HT (Fig. 9C; F _(1,60)_ = 41.07, *P* < 0.0001), but not 5-HT_7_ receptor agonist LP44 (Fig. 9D; F_(1,48)_ = 3.308, *P* = 0.0752), reduced the latency of tail-flick in response to 52 °C hot water in BDL rats. In contrast, as shown in Fig. 9A–D, only 5-HT_2_ receptor agonist α-methyl-5-HT significantly reduced the latency of tail-flick in response to 52 °C hot water (F_(1, 55)_ = 8.913, *P* = 0.0042) in sham rats,, but 5-HT_1A_ receptor agonist DPAT (F_(1,96)_ = 0.6788, *P* = 0.4120), 5-HT_3_ receptor agonist 2-Methyl-5-HT (F_(1,55)_ = 0.8980, *P* = 0.3475), and 5-HT_7_ receptor agonist LP44 (F_(1,54)_ = 0.3522, *P* = 0.5553) had no effects. Thus, it was suggested that over-activation of 5-HT receptors 5-HT_1A_, 5-HT_2_, and 5-HT_3_ might attenuate antinociception in BDL rats.

We next assessed the effects of intrathecal (i.t.) or intraperitoneal (i.p.) injection of 5-HT receptors antagonists on antinociception in sham and BDL rats after BDL surgery 15 days. The results showed that i.t. injection of 5-HT_1A_ receptor antagonist WAY-100635 significantly increased the latency of tail-flick in response to 52 °C hot water in sham and BDL rats (Fig. 9E; for sham: F_(1,72)_ = 95.79, *P* < 0.0001; for BDL: F(1, 60) = 7.657, *P* = 0.0075). Interestingly, i.p. application of 5-HT_2_ receptor antagonist ketanserin (Fig. 9F) and 5-HT_3A_ receptor antagonist ondansetron (Fig. 9G;) significantly reduced the latency of tail-flick in response to 52 °C hot water in BDL rats (for ketanserin: F_(1,60)_ = 42.43, *P* < 0.0001; for ondansetron: F_(1, 50)_ = 24.14, *P* < 0.0001), but not in sham rats (for ketanserin: F_(1, 48)_ = 3.409, *P* = 0.0710; for ondansetron: F_(1, 40)_ = 0.8852, *P* = 0.3524). Finally, i.t. injection of 5-HT_7_ receptor antagonist SB269970 failed to change the latency of tail-flick in response to 52 °C hot water in sham and BDL rats (Fig. 9H; for sham: F_(1,48)_ = 0.01346, *P* = 0.9081; for BDL: F_(1,60)_ = 1.261, P = 0.2660). Thus, it was suggested that antagonism of 5-HT_1A_ might produce antinociception in sham and BDL rats, while antagonism of 5-HT_2_ and 5-HT_3_ might attenuate antinociception in BDL rats. Additionally, i.t. injection of 5-HT_7_ receptor antagonist SB269970 had little effects on antinociception in sham and BDL rats.

## Discussion

Several clinical observation showed that administration of 5-HT_3_ receptor antagonist ondansetron^34^,^35^,^36^,^37^,^38^ or selective serotonin reuptake inhibitor (SSRI) sertraline^39^,^40^ were able to alleviate cholestatic pruritus, providing important clues to support key role of 5-HT system in cholestatic pruritus. Unfortunately, little is known of the modulatory effects of peripheral and central 5-HT system, especially 5-HT receptor subtypes, on cholestatic pruritus and antinociception so far^73^,^74^. Based on the important and complex roles of 5-HT system in the regulation of pain and itch^75^, the present study investigated the roles of peripheral and central 5-HT system in itch and antinociception in BDL rats, which is a severe model of obstructive cholestasis^42^. Our results revealed that peripheral and central 5-HT system played important roles in modulation of itch and antinociception in BDL rats. Although no obvious spontaneous scratching was observed in BDL rats, we found an enhanced scratching response was induced by i.d. injection of 5-HT in BDL rats, suggesting itch hypersensitivity in BDL rats. We further showed that the 5-HT level in the skin and spinal cord significantly increased, possible due to the increased 5-HT synthesis, in BDL rats compared to sham rats. In BDL rats, several 5-HT receptor subtypes were up-regulated in the DRG or TG, including 5-HT_1B_, 5-HT_1D_, 5-HT_2A_, 5-HT_3_, 5-HT_5B_, 5-HT_6_, and 5-HT_7_. In sharp contrast, multiple 5-HT receptor subtypes were down-regulated in the spinal cord or brainstem in BDL rats, including 5-HT_1A_, 5-HT_1F_, 5-HT_2B_, and 5-HT_3_. Pharmacological activation of 5-HT_2A_, 5-HT_3_, and 5-HT_7_ induced itch in BDL rats. Administration of 5-HT_1A_, 5-HT_2A_, 5-HT_3_, and 5-HT_7_ agonists or antagonists differentially influenced the itch and antinociception in sham and BDL rats. Thus, these results suggested 5-HT system dynamically participated in itch and antinociception under cholestasis condition and distinct 5-HT receptor subtypes might be involved in these processes.

There are several animal models to mimic cholestasis-induced itch and antinociception, including BDL in rats^20^,^24^ or mice^21^, administration of α-naphthylisothiocyanate (ANIT) or 17α-ethynylestradiol in rats^76^ or mice^77^. Interestingly, we noticed that spontaneous scratching behavior was observed in BDL rats^24^ and in 17α-ethynylestradiol-treated rats^76^; However, impaired itch perception was also demonstrated in ANIT or 17α-ethynylestradiol-treated mice^77^. In the current study, we employed BDL rat model to investigate itch and antinociception associated with severe obstructive cholestasis. We carefully quantified the spontaneous itch-related behaviors, such as scratching, grooming, and biting behaviors after BDL surgery in SD rats^24^. Surprisingly, we found itch-related behaviors were not significantly increased in BDL rats compared to sham rats. We postulated the discrepancy for spontaneous itch in BDL rats may be attributed to the different rat stains employed: SD rats versus Wistar rats. Although lack of spontaneous scratching behavior in BDL rats, we observed an enhanced scratching induced by intradermal injection of 5-HT in BDL rats, suggesting evoked itch was potentiated in cholestatic rats. Thus, species difference among mice, rats and human and the etiology of cholestasis may explain the distinct itch phenotype under cholestasis condition. After BDL surgery, rats developed antinociception responding to mechanical or heat stimuli, which were consistent with others’ reports^20^,^42^,^78^. In addition, the motor performance of BDL rat was intact evaluated by Rota-rod test, suggesting that itch and antinociception phenotypes of BDL rats may not be attributed to motor dysfunction.

Patients with cholestatic pruritus also showed increased number of dermal mast cells, which release histamine, 5-HT and proteases^24^. Our immunostaining data and HPLC analysis provided evidence that 5-HT level in the skin and the spinal cord significantly increased in BDL rats compared to sham rats. We further found the mRNA expression of *Tph1* and *Tph2*, the rate-limiting enzymes for 5-HT synthesis in the skin and in the brain stem, respectively^26^, were up-regulated in BDL rats. Thus, it suggested that increased 5-HT level may be attributed to enhanced 5-HT synthesis. Because histamine seems to play little role in cholestasis pruritus^79^, our data suggested 5-HT, possible from mast cells, may serve as potential itch mediator under cholestatais condition.

We subsequently demonstrated that intradermal injection 5-HT_2A_, 5-HT_3_ or 5-HT_7_ receptor agonists induced scratching in BDL rats, whereas only 5-HT_2A_, or 5-HT_7_ receptor agonists induced scratching in the sham rats, suggesting 5-HT_3_ receptor is involved in pathological cholestatic itch but not physiological itch. Our results also demonstrated application of 5-HT_3_ receptor antagonist ondansetron attenuated itch in BDL rats but not in sham rats. It was consistent with the clinical observation that 5-HT_3_ receptor antagonist ondansetron alleviated cholestatic pruritus^34^. We further showed that DCA was not able to induce scratching behavior in rats, although it induced itch in mice^17^,^23^, suggesting the species difference between rat and mouse existed for bile acid-induced itch. Interestingly, our results showed that BDL rats did not scratch spontaneously but exerted enhanced scratch induced by 5-HT. These data was consistent with the observation that DCA did not evoke scratch in rats, but potentiated 5-HT-induced scratch in rats. Together, these data suggested 5-HT, possible not bile acids, may serve as a potential pruritogen in rats under cholestasis condition. Both increased 5-HT level in skin and increased expression of 5-HT_2A_, 5-HT_3_ or 5-HT_7_ receptors in peripheral nervous system contributed to itch hypersensitivity under cholestasis condition. However, the roles of other 5-HT receptors subtypes, such as 5-HT_1D_, 5-HT_5B_, and 5-HT_6_, on cholestatic itch remain unclear and warrant further investigation.

As we demonstrated 5-HT-induced itch was enhanced in BDL rats, we subsequently investigated the dynamic expression changes of 5-HT receptors subtypes in peripheral and central nervous systems by using qPCR analysis under cholestasis condition. The results demonstrated that mRNA expression of multiple 5-HT receptors, including 5-HT_1B_, 5-HT_1D_, 5-HT_2A_, 5-HT_3A_, 5-HT_5B_, 5-HT_6_, and 5-HT_7_, were up-regulated in DRG or TG from BDL rats compared to sham rats. In sharp contrast, the mRNA expression of multiple 5-HT receptors, including 5-HT_1A_, 5-HT_1F_, 5-HT_2B_, and 5-HT_3A_, were down-regulated in spinal cord or brainstem (especially cervicomedullary junction) from BDL rats compared to that from sham rats. As we showed increased 5-HT level in CNS could suppress 5-HT-induced scratch in sham and BDL rats, the down-regulation of 5-HT receptors may produce dis-inhibition to contribute to the enhanced itch in BDL rats. Thus, these results suggested up-regulation of certain 5-HT receptors in primary sensory neurons and down-regulation of certain 5-HT receptors in central nervous system may contribute to the enhanced itch behavior in BDL rats. It is unclear for the precise causes to drive the expression changes of 5-HT receptors in BDL rats.

As 5-HT is not able to penetrate blood-brain-barrier (BBB), peripheral and central 5-HT systems are considered as two separated compartments^25^ and the synthesis of 5-HT also employs distinct TPH, a rate-limiting enzymes for 5-HT synthesis. To investigate the possible role of central 5-HT in cholestatic itch, we performed i.t. injection 5-HT or i.p. injection of selective serotonin reuptake inhibitor (SSRI) fluoxetine in the sham and BDL rats. It was found that 5-HT (i.t.) or fluoxetine (i.p.) significantly suppressed 5-HT-induced scratching in sham and BDL rats, suggesting increased 5-HT level in CNS is sufficient to suppress itch in sham and BDL rats. We further used pharmacological 5-HT receptor agonists to reveal the distinct role of certain 5-HT receptor subtypes that involved in. Our results showed that intrathecal injection of 5-HT_1A_, 5-HT_2A_, 5-HT_3_ or 5-HT_7_ receptors agonists suppressed 5-HT-induced scratching in sham and BDL rats, which was consistent with previous report^80^. Thus, the results suggested application 5-HT or 5-HT receptor agonists, including 5-HT_1A_, 5-HT_2A_, 5-HT_3_ or 5-HT_7_, inhibited itch sensation possible through over-activation of these 5-HT receptors subtypes in the spinal cord. We next examined whether increased endogenous 5-HT level in spinal cord contributed to itch hypersensitivity in BDL rats by using selective 5-HT receptor antagonists. Our results showed that antagonism of 5-HT_1A_, 5-HT_2A_, 5-HT_3_ or 5-HT_7_, inhibited itch sensation in BDL rats, suggested increased endogenous 5-HT in spinal cord of BDL rats might play an itch-facilitating effect through 5-HT_1A_, 5-HT_2A_, 5-HT_3_ or 5-HT_7_. In sham control rats, antagonism of 5-HT_1A_ increased, while antagonism of 5-HT_2A_, or 5-HT_7_ inhibited itch. Meanwhile, antagonism of 5-HT_3_ had little effect on itch in sham rats. Thus, it was suggested tonic activation of spinal 5-HT_1A_ might play a suppressive effect, while activation of 5-HT_2A_ and 5-HT_7_ might play a facilitating effect on itch transmission in physiological condition.

What is the role of spinal 5-HT_1A_ in regulating cholestatic pruritus? Our results showed 5-HT_1A_ antagonist exerted itch-enhancing effect in sham rats but alleviated itch in BDL rats. Previous study demonstrated that descending serotonergic system from brainstem facilitated gastrin-releasing peptide (GRP)-GRP receptor (GRPR) signaling via spinal 5-HT_1A_ for mediating itch transmision. In sharp contrast, activation of 5-HT_1A_ hyperpolarizes spinal neurons without GRPR to dampen neuronal excitability, suggesting opposing modulation of descending serotonergic system on itch and pain. The dual actions of 5-HT mediated by 5-HT_1A_ suggested neuronal phenotypes (excitatory versus inhibitory) that expresses this receptor might play a key role in determining which actions 5-HT would finally exert on itch neurotransmission. Whether the down-regulation of 5-HT_1A_, possible other subtypes 5-HT_1F,_ 5-HT_2B_ and 5-HT_3A_, in spinal cord contributes to itch hypersensitivity in BDL rats warrants further study.

To investigate the possible role of spinal 5-HT in cholestasis-associated antinociception, we performed i.t. injection of 5-HT or i.p. injection of selective serotonin reuptake inhibitor (SSRI) fluoxetine in the sham and BDL rats. 5-HT (i.t.) or fluoxetine (i.p.) significantly increased the heat pain threshold in sham and BDL rats, suggesting increased 5-HT level in spinal cord might be sufficient to induced antinociception in sham and BDL rats, which was consistent with previous report^81^. We next used selective 5-HT receptors agonist to investigate the distinct role of certain 5-HT receptor subtypes that involved in. Our results showed that i.t. injection of 5-HT_1A_, 5-HT_2A_, and 5-HT_3_, but not 5-HT_7_ receptor agonists suppressed antinociception in BDL rats. Thus, the results suggested over-activation of 5-HT_1A_, 5-HT_2A_, and 5-HT_3_ attenuated antinociception under cholestasis condition. We next examined whether increased endogenous 5-HT level in spinal cord contributed to antinociception in BDL rats by using selective 5-HT receptors antagonists. Our results showed that antagonism of 5-HT_2A_ and 5-HT_3_ inhibited antinociception in BDL rats, suggesting increased endogenous 5-HT in spinal cord of BDL rats exert antinociception possible through 5-HT_2A_ and 5-HT_3_. Antagonism of 5-HT_1A_ increased heat pain threshold in both sham and BDL rats, suggesting activation spinal 5-HT_1A_ might play a pronociceptive role in sham and BDL rats. Additionally, agonism or antagonism of 5-HT_7_ had little effect on antinociception in sham and BDL rats. The limitation of our pharmacological study is the lack of dose-response curves for different 5-HT receptor agonists or antagonists. Thus, our results suggested activation of spinal 5-HT_1A_ might play a pronociceptive role, while activation of 5-HT_2A_ and 5-HT_3_ might play an antinociceptive role in BDL rats.

Previous reports have proposed the endogenous opioidergic system involved in itch and antinociception under cholestasis condition^75^. Our results suggested serotonergic system was also important for modulating cholestasis-induced itch and antinociception. Endogenous opioids-induced analgesic effects partially mediated by activation of descending serotonergic inhibitory pathways terminating on spinal cord dorsal horn^31^. 5-HT_1A_ receptor acts as a regulator for 5-HT release, its down-regulation could increase 5-HT release^82^. Previous report demonstrated the expression of 5-HT_1A_ was down-regulated in hippocampus of BDL mice^82^. Our results also showed the expression 5-HT_1A_ was down-regulated in spinal cord and brainstem in BDL rats, which may also contribute to the increased release of 5-HT.

In summary, our findings showed that itch hypersensitivity and antinociception developed in BDL rats. We found that 5-HT level increased in the skin and spinal cord in BDL rats. 5-HT receptors subtypes, including 5-HT_1B_, 5-HT_1D_, 5-HT_2A_, 5-HT_3A_, 5-HT_5B_, 5-HT_6_, and 5-HT_7,_ were up-regulated in peripheral nervous system and 5-HT_1A_, 5-HT_1F_, 5-HT_2B_, and 5-HT_3A_ were down-regulated in central nervous system. Peripheral activation of 5-HT_2_, 5-HT_3_, and 5-HT_7_ might contribute to cholestatic itch in rats. Agonism or antagonism of 5-HT receptors 5-HT_1A_, 5-HT_2A_, 5-HT_3_, and 5-HT_7_ might suppress cholestatic itch. Agonism or antagonism of 5-HT receptors 5-HT_1A_, 5-HT_2A_, and 5-HT_3_ differently modulate antinociception in BDL rats. Thus, targeting 5-HT system may provide an effective treatment for cholestatic pruritus and possible other comorbidities.

## Additional Information

**How to cite this article**: Tian, B. *et al*. Peripheral and spinal 5-HT receptors participate in cholestatic itch and antinociception induced by bile duct ligation in rats. *Sci. Rep.* **6**, 36286; doi: 10.1038/srep36286 (2016).

**Publisher’s note:** Springer Nature remains neutral with regard to jurisdictional claims in published maps and institutional affiliations.

## Figures and Tables

**Table 1:** 5-HT receptor agonists and antagonists used in this study.

Chemicals	Target	Formula	M.Wt	Biological Activity	Ref.
DPAT	5-HT_1A_ receptor agonist	C_16_H_25_NO.HBr	328.29	Full 5-HT_1A_ serotonin receptor agonist; more active enantiomer. Reduces hippocampal 5-HT levels following systemic administration in rats *in vivo*.	44
WAY-100635	5-HT_1A_ receptor antagonist	C_25_H_34_N_4_O_2_.C_4_H_4_O_4_	538.64	Potent, silent antagonist of 5-HT_1A_ receptors (IC50 = 2.2 nM; Ki = 0.84 nM for rat 5-HT_1A_ receptors). Displays 100-fold selectivity for 5-HT_1A_ over other 5-HT subtypes.	45, 46
α-methyl-5-HT	5-HT_2_ receptor agonist	C_11_H_14_N_2_O · C_4_H_4_O_4_	306.31	a-methyl-5-HT displays a high affinity (Ki = 3 nM with [^3^H]DOB) for 5-HT_2_ site and little selectivity for 5-HT_1A_, 5-HT_1B_, 5-HT_1C_, and 5-HT_1D_ sites (Ki = 42, 85, 150, and 150 nM, respectively) and a very low affinity for 5-HT_1E_ (Ki greater than 10,000 nM) sites.	47, 48
ketanserin	5-HT_2A_ receptor antagonist	C_22_H_22_FN_3_O_3_ · C_4_H_6_O_6_	545.51	Selective 5-HT_2_ serotonin receptor antagonist. Ketanserin significantly reduces nicotine self-administration in rats, supporting an unexpected involvement of serotonin in nicotine addiction.	49, 50
2-Methyl-5-HT	5-HT_3A_ receptor agonist	C_11_H_14_N_2_O.HCl	226.71	5-HT_3_ agonist (K_i_ = 1200 nM) and potent 5-HT_6_ ligand (K_i_ = 46 nM).	51, 52
Ondansetron	5-HT_3A_ receptor antagonist	C_18_H_19_N_3_O.HCl	329.83	Selective 5-HT_3_ receptor antagonist (K_i_ = 6.16 nM). Antiemetic; prevents emesis induced by cytotoxic drugs and radiation.	53, 54, 55
LP44	5-HT_7_ receptor agonist	C_27_H_37_N_3_OS.HCl	488.13	High affinity 5-HT_7_ receptor agonist (K_i_ = 0.22 nM) that displays selectivity over 5-HT_1A_ and 5-HT_2A_ receptors (200- and >1000-fold respectively). Induces relaxation of substance P-stimulated guinea pig ileum (EC_50_ = 2.56 μM).	29, 56
SB 269970	5-HT_7_ receptor antagonist	C _18_H_28_N_2_O_3_S.HCl	388.95	Potent and selective 5-HT_7_ receptor antagonist (pK_i_ values are 8.9, 7.2 and 6.0 for 5-HT_7A_, 5-HT_5A_ and 5-HT_1B_ and < 6.0 for 5-HT_1A_, 5-HT_1D_, 5-HT_1E_, 5-HT_1F_, 5-HT_2A_, 5-HT_2B_, 5-HT_2C_, 5-HT_4_ and 5-HT_6_ receptors respectively).	29, 57, 58

**Table 2:** Sequences of primers used for real-time quantitative PCR.

Target gene	Forward (5′-3′)	Reverse (5′-3′)	Product size (bp)	Accession number
5-HT_1A_	CCATCAGCAAGGACCACGGCTA	CCCGTAGAGAACCAGCATGAGCAA	86	NM_012585.1
5-HT_1B_	GTCAAGCCAAAGCGGAGGA	GCAGGGTGGGTAAATAGAAAGC	105	NM_022225.1
5-HT_1D_	CCCGAGAAAGGAAAGCCACT	GAGGACCAAGGATACCACAAAGAA	92	NM_012852.1
5-HT_1F_	CTGTGACCTTTGGCTGAGTGTT	CGACTGCGTCTGTGATTGCTC	104	NM_021857.3
5-HT_2A_	CTTCCAACGGTCCATCCACA	GGGCACCACATTACAACAAACAG	132	NM_017254.1
5-HT_2B_	CGCCATCCCAGTCCCTATT	CAGCCAGTGACCCAAAGAGC	116	NM_017250.1
5-HT_2C_	GACTGAGGGACGAAAGCAAAG	GAAGGACCCGATGAGAACGA	83	NM_012765.3
5-HT_3A_	GTGACCGCCTGTAGCCTTGA	GATGCTCTTGTCCGACCTCA	147	NM_024394.2
5-HT_4_	TGCCTTCCTTATCATCCTCTGC	CACCACATTCCACTGTATCCCT	134	NM_012853.1
5-HT_5A_	CGCTGTGCTCCTGGGATAT	CCTGTTGAACGCCGTGTAGAT	104	NM_013148.1
5-HT_5B_	CGTGGTGCTCTTCGTCTACTG	TCCTGAGGTGCTTCCTTTGC	117	NM_024395.1
5-HT_6_	GCACGAACTGGGCAAAGCT	GGACGCCACGAGGACAAAA	82	NM_024365.2
5-HT_7_	TTCTGTCGGTCTGGCTGCTCTC	ACCGCAGTGGAGTAGATCGTGTAG	130	NM_022938.2
CK-7	CGAGGAGATGGCCAACCATA	GAGCGGTTCATCTCCGCAAT	138	NM_001047870.1
PCNA	CTTACTCTGCGCTCCGAAGG	TGATGTCTTCATTACCAGCACA	115	NM_022381.3
TPH1	ACGAACTCTTAGGCCACGTCC	TTGCACAGTCCAAACTCCACA	151	NM_001100634.2
TPH2	CAGCCCGCAATGATGATGTTT	CGCTTCTCTTGTCCTCGCTTT	145	NM_173839.2
Actin	ACTATCGGCAATGAGCGGTTCC	AGCACTGTGTTGGCA TAGAGGTC	152	NM_031144.3
